# DAPK1 Signaling Pathways in Stroke: from Mechanisms to Therapies

**DOI:** 10.1007/s12035-016-0008-y

**Published:** 2016-07-22

**Authors:** Shan Wang, Xiangde Shi, Hao Li, Pei Pang, Lei Pei, Huiyong Shen, Youming Lu

**Affiliations:** 1 Department of Biotherapy Technology Center, Sun Yat-sen Memorial Hospital, Sun Yat-sen University, Guangzhou, 510120 China; 2 Department of Biliary Pancreatic Surgery, Sun Yat-sen Memorial Hospital, Sun Yat-sen University, Guangzhou, 510120 China; 3 Department of Physiology, School of Basic Medicine, Tongji Medical College, Huazhong University of Science and Technology, Wuhan, 430030 China; 4 The Institute for Brain Research (IBR), Collaborative Innovation Center for Brain Science, Huazhong University of Science and Technology, Wuhan, 430030 China; 5 Department of Neurobiology, School of Basic Medicine, Tongji Medical College, Huazhong University of Science and Technology, Wuhan, 430030 China; 6 Department of Orthopedics, Sun Yat-sen Memorial Hospital, Sun Yat-sen University, Guangzhou, 510120 China

**Keywords:** DAPK1, Stroke, Cell death, Mechanism, Therapeutics

## Abstract

Death-associated protein kinase 1 (DAPK1), a Ca^2+^/calmodulin (CaM)-dependent serine/threonine protein kinase, plays important roles in diverse apoptosis pathways not only in tumor suppression but also in neuronal cell death. The requirement of DAPK1 catalytic activity for its proposed cell functions and the elevation of catalytic activity of DAPK1 in injured neurons in models of neurological diseases, such as ischemia and epilepsy, validate that DAPK1 can be taken as a potential therapeutic target in these diseases. Recent studies show that DAPK1-NR2B, DAPK1-DANGER, DAPK1-p53, and DAPK1-Tau are currently known pathways in stroke-induced cell death, and blocking these cascades in an acute treatment effectively reduces neuronal loss. In this review, we focus on the role of DAPK1 in neuronal cell death after stroke. We hope to provide exhaustive summaries of relevant studies on DAPK1 signals involved in stroke damage. Therefore, disrupting DAPK1-relevant cell death pathway could be considered as a promising therapeutic approach in stroke.

## Introduction

Stroke, a major cause of morbidity and mortality, affects millions of lives worldwide every year [1]. It is either due to cerebral ischemia or hemorrhage and is followed with a series of complex biochemical incidents that leads to the total breakdown of cellular integrity and eventually cell death. Accumulative evidence suggests that ischemic stroke-induced neuronal cell death is likely due to excitotoxicity for an excessive stimulation of glutamate receptors [2–4] resulting in Ca^2+^ overloading, oxidative radical stress involved in reactive oxygen species (ROS)/reactive nitrogen species (RNS) production [2, 5–7], and suicidal events such as apoptosis and necrosis [5, 6, 8, 9]. Blocking the glutamate receptors after ischemic insults has been shown to both be effective [1] and have severe side effects in stroke therapy [10]. Therefore, glutamate receptors are not desired targets in preventing ischemic neuronal death. Spectacular failures in drug development programs and clinical trials for neuroprotective agents have led to the withdrawal of funding aimed at developing new drugs for stroke [1]. The direct and effective treatments for stroke remain lacking other than reopening an occluded artery with thrombolytic drugs, which makes the identification of new therapeutic targets a matter of great importance.

Death-associated protein kinase 1(DAPK1), identified in a screen for genes that influence γ-interferon (IFN)-induced cell death in HeLa cells [11], is a 160-kDa Ca^2+^/calmodulin-dependent serine/threonine protein kinase [12] whose phosphorylation activity is known to be responsible for certain forms of apoptotic cell death, including Fas, tumor necrosis factor (TNF)-α [13], ceramide [14], caspase [15], and p53-mediated apoptosis [16], as well as in the disruption of matrix survival signals and suppression of integrin-mediated cell adhesion [17]. In addition, DAPK1, which is abundantly expressed in the brain, has been linked to neurological diseases associated with neuronal injury and may serve as a target for therapeutic intervention in the treatment of stroke, epilepsy, and Alzheimer’s disease. DAPK1 is of particular interest in stroke to us because of a quantitative proteomic analysis of the death-signaling proteins that are enrolled to the cytoplasmic tail of the N-methyl-D-aspartate receptor (NMDAR) during cerebral ischemia revealing DAPK1 as the most prevalent protein [18]. Over the past decades, several groups have made efforts to decipher DAPK1’s cellular function in stroke, focusing on its biochemical properties, regulation, and especially the target substrates in ischemic injuries. It has become apparent that DAPK1 has multiple roles and is linked to several cell death-related signaling pathways in ischemia. The purposes of this review are to provide a comprehensive overview of the recent literatures on DAPK1 signals in ischemic stroke and to help us better understand the molecular mechanisms of neuronal cell death during stroke injuries.

## A General Introduction of DAPK1

**Fig. 1:**
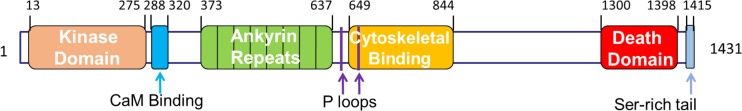
Schematic diagram of human-derived DAPK1 protein structure. Shown are the known functional domains of human DAPK1. See text for details

DAPK1 has a unique multidomain structure containing an N-terminal kinase domain, followed by a calmodulin (CaM) regulatory segment, eight ankyrin repeats, a cytoskeleton binding region, two P-loop motifs, and a C-terminal death domain as well as a stretch of amino acids that are rich in serines and threonines [19] (Fig. 1). DAPK1 belongs to a family of related death protein kinases, which consists of other two closely related homologs: ZIP kinase (ZIPK, also known as DAP-like kinase (Dlk) or DAPK3) [20, 21] and DAPK-related protein 1 (DRP-1, also known as DAPK2) [22, 23], whose human genes share 83 and 80 % of the identity of amino acids, respectively, with DAPK1’s kinase domain. The death-promoting effects of DAPK1 mostly depend on its catalytic activity which is under tight control, to ensure on one hand its silence under normal growth conditions, and to allow, on the other hand, rapid activation in response to the appropriate apoptotic signal.

The identification of myosin light chain (MLC) as a substrate of DAPK1 facilitated the performance of in vitro DAPK1 kinase assays which enables the analysis of different aspects of its catalytic activity and of its mode of regulation [24]. DAPK1 is regulated by a number of mechanisms. Firstly, the CaM-regulatory segment, which acts as a pseudosubstrate to the cleft of kinase domain, possesses an auto-inhibitory effect on the catalytic activity and can be relieved by binding to Ca^2+^-activated CaM [25] and therefore activates DAPK1. Second, DAPK1 is negatively regulated by autophosphorylation on serine 308 in the Ca^2+^/CaM regulatory domain at the basal level. Dephosphorylation relieves autoinhibition, enhances the interaction between CaM and the DAPK1 CaM-regulation segment, and stimulates its proapoptotic activities [25]. Consistently, the mere deletion of this segment from DAPK (ΔCaM) or the mutation of Ser308 to alanine (S308A) generates a constitutively active kinase.

The catalytic activity of DAPK1 is controlled by distinct mechanisms. First, DAPK1 loses catalytic activity upon mutation of Lys42 to Ala (K42A) [19], one of the amino acids critical to the binding with ATP [26]. Second, extracellular-regulated protein kinase1/2 (ERK1/2) binds a docking site within DAPK1’s death domain and phosphorylates DAPK1 Ser735 within the cytoskeletal binding region both in vitro and in vivo, stimulating DAPK1 catalytic activity [27]. Third, the p90 ribosomal S6 kinase (RSK), a downstream effector of ERK, inhibits exogenous DAPK1 cellular activity by phosphorylation of Ser289 within the CaM-autoregulatory/binding segment [28]. Last but not least, DANGER, denoted by Damian B et al., inhibits DAPK1 activity toward MLC in a concentration-dependent manner, without influencing calmodulin’s binding to DAPK1 [29]. Then, how is DAPK1 activated during ischemic stroke? It is known that cerebral ischemia induces overexcitation of the NMDA receptor, causing excessive Ca^2+^ flow into the cytoplasm and activates not only CaM but also the calcineurin phosphatase (CaN) [30] and dephosphorylates DAPK1 in Ser308. Then, DAPK1 combines with CaM and becomes activated.

## DAPK1 and its Kinase Activity in Ischemic Neuronal Death

In the developing and adult central nervous system, DAPK1 mRNA is widely expressed in proliferative regions within the cerebral cortex and hippocampus [31, 32]. In addition, DAPK1 is critically involved in the processes of both neuronal development and recovery from injury, as its activity is increased in response to hypoxic ischemia [24, 33]. The temporal and spatial pattern of regulation suggests an important role of DAPK1 in neuronal functions. The expression of DAPK1 mRNA is increased prior to selective cell death induced by transient forebrain ischemia, indicating a close relationship between DAPK1 and neuronal cell death [32]. Moreover, a small molecule inhibitor of DAPK, alkylated 3-amino-6-phenylpyridazine, significantly attenuates brain injury after ischemic stroke [34]. In addition, the activation of DAPK1 has also been implicated in seizure-induced neuronal death [35, 36].

The cell death-inducing activation of DAPK1 largely depends on its intrinsic kinase activity [19]. For example, the overexpression of intact DAPK1, but not of the catalytically inactive kinase mutant in HeLa cells, induces apoptotic cell death. It has been demonstrated that DAPK1 phosphorylates diverse substrates via its kinase domain. These substrates include the MLC [19], beclin-1 [37, 38], zipper-interacting protein kinase (ZIPK) [39, 40], calmodulin-regulated protein kinase kinase (CaMKK) [41], and syntaxin-1A [42]. The requirement of DAPK catalytic activity for its proposed cell functions and the validation of protein kinases as therapeutic targets in human disease make the identification of substrates of DAPK1 in ischemia extremely important.

### DAPK1 and NR2B

During stroke, energy failure occurs and ionic gradients are lost, then glutamate is released accompanied by impaired reuptake processes, and this redundant excitatory amino acid binds to its postsynaptic receptors and also leaks out to the extrasynaptic receptor and promotes excessive calcium entry and calcium release, triggering neuronal death signaling. N-methyl-D-aspartate receptors (NMDARs) are cation channels that are gated by glutamate in the brain. Like a double-edged sword, NMDARs play very important roles in both neuronal health and neuronal death. It is known that NMDAR-induced responses depend on the receptor location and subunit constitution. For example, the activation of synaptic NMDARs, mainly NR1 and NR2A, acting primarily through nuclear Ca^2+^ signaling, leads to the build-up of a neuroprotective “shield” [43], whereas stimulation of extrasynaptic NMDARs (NR2B) promotes neuronal cell death [44].

**Fig. 2:**
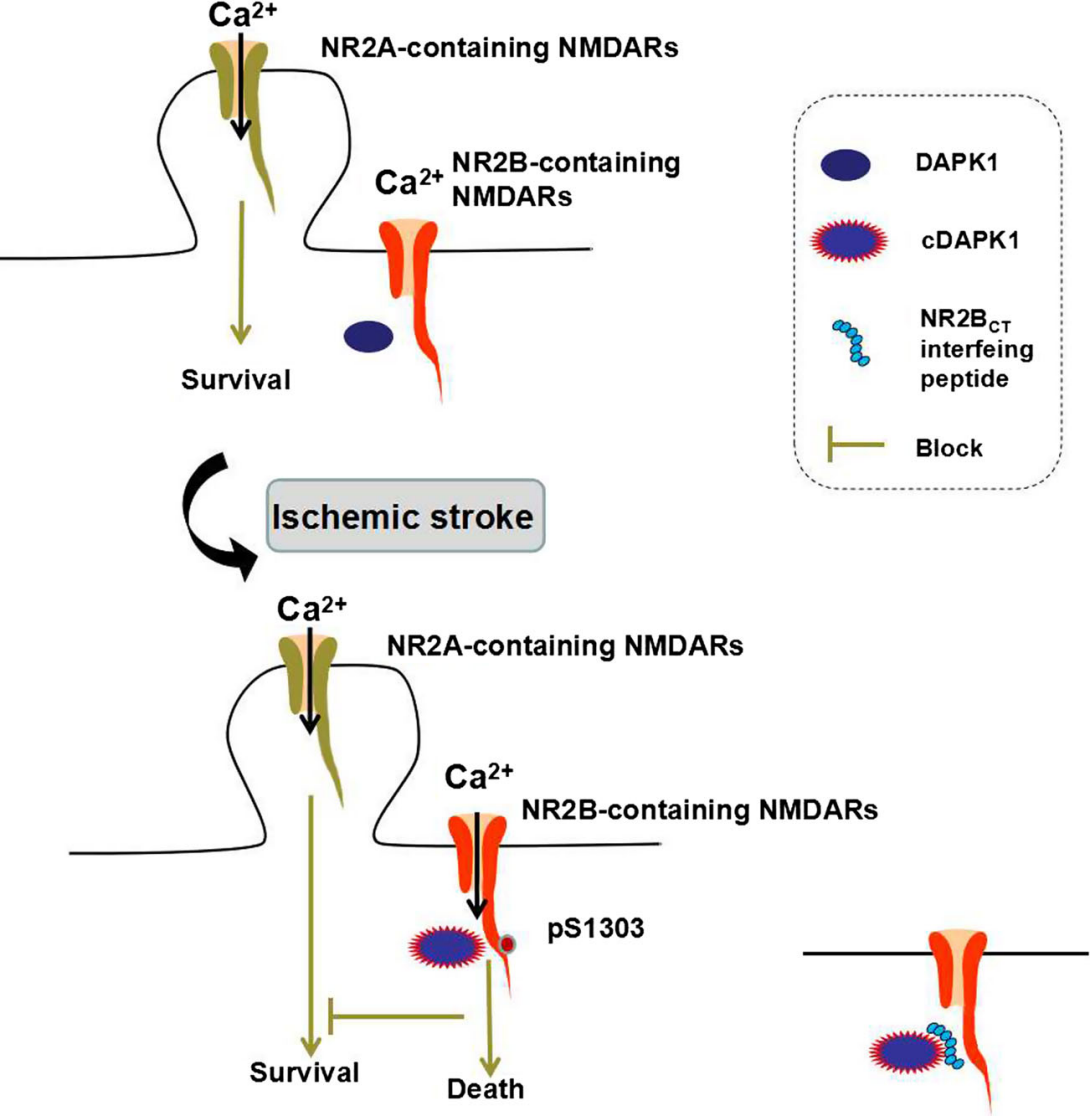
Illustration for DAPK1-NR2B. DAPK1 is inactive in physiological state. After cerebral ischemic stroke, DAPK1 is activated (cDAPK1, constitutively active DAPK1) and combines with the extrasynaptic NMDARs, phosphorylating the serine 1303 in the NR2B C-terminal (CT) tail and mediating cell death. Blocking DAPK1-NMDARs interaction with an NR2B_CT_-interfering peptide resists ischemic stroke damage

A study by Tu et al. reported that DAPK1 is directly linked to the NR2B subunit and initiates a specific cell death signaling [18]. They identified that DAPK1 directly binds with the carboxyl tail region consisting of amino acid 1292–1304 (NR2B^CT^) of the NR2B subunit. A constitutively active DAPK1 phosphorylates the NR2B subunit at Ser-1303 and in turn enhances the NR1/NR2B receptor channel conductance. The administration of a peptide NR2B^CT1292–1304^ to uncouple of the activated DAPK1 from the NMDA receptor complex protects against brain damage in stroke without affecting the physiological actions of the NMDA receptors (Fig. 2). Thus, targeting DAPK1-NMDA receptor interaction can be considered as a practical strategy for stroke therapy [45].

### DAPK1 and p53

**Fig. 3:**
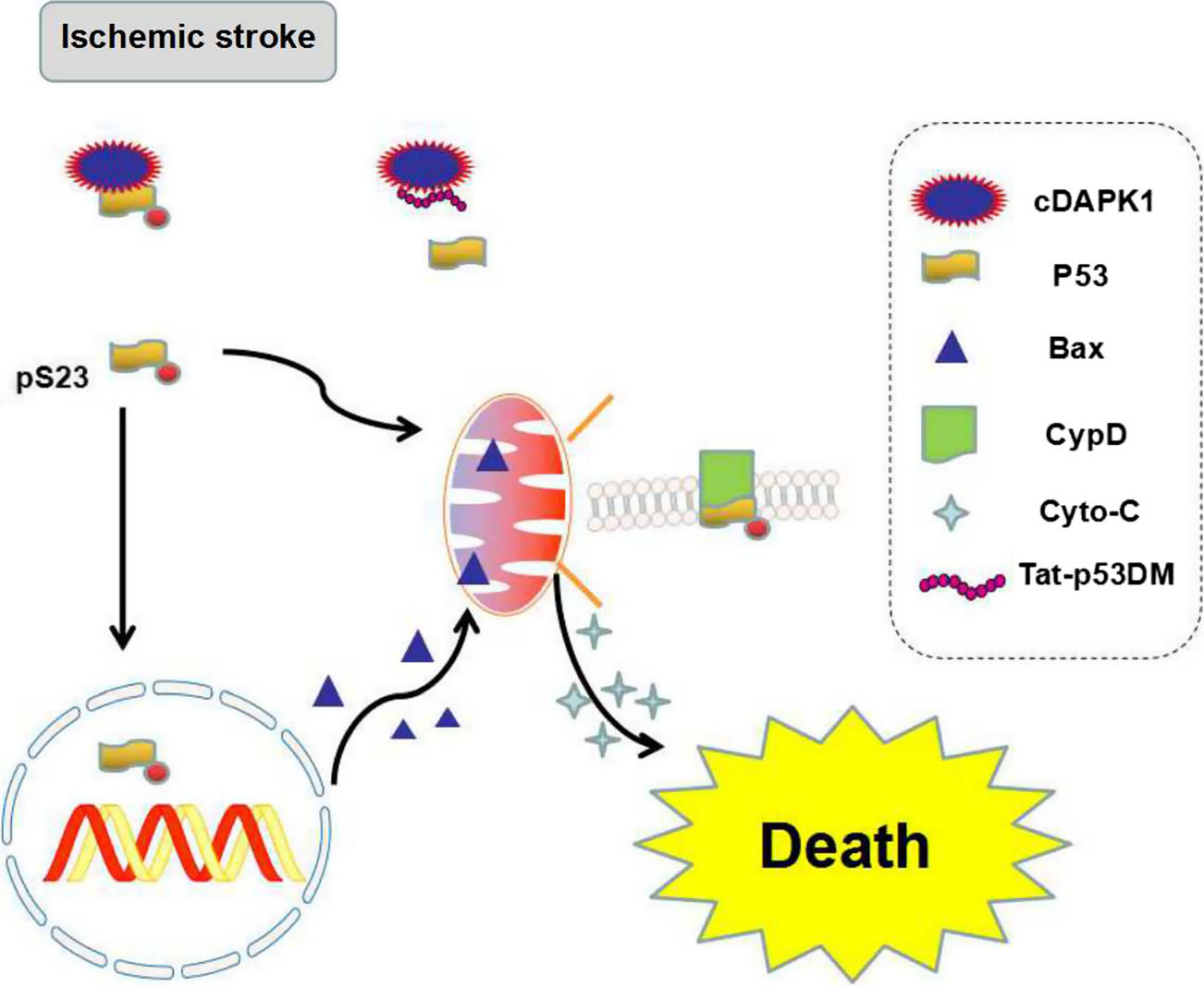
Illustration for DAPK1-p53. The interaction of DAPK1–p53 activates both necrotic and apoptotic signaling through transcription- and mitochondria-dependent pathways. DAPK1 binds to p53DM and phosphorylates p53 at serine 23 (pS23), which on one hand translocates into the nucleus and activates the proapoptotic gene expression and apoptosis. On the other hand, the pS23 also enters into the mitochondrial matrix and interacts with CypD and induces necrosis. A peptide Tat-p53DM blocks the interaction of DAPK1 and p53 effectively (modified from Pei et al., The Journal of Neuroscience, 2014. 34(19): p. 6546–56 )

Tumor suppressor gene p53 is a transcriptional regulator and controls the program of necrotic and apoptotic pathways of cell death involved in the neurodegeneration progress including ischemic stroke, epilepsy, and Alzheimer’s disease through transcriptional-dependent and transcriptional-independent mechanisms [46–48]. DAPK1 is considered a kinase of human p53 at serine-20 (pS20), a residue corresponding to mouse p53 at serine-23 (pS23, 19QETFSGL25) [49]. It has been reported that DAPK1 might interact with p53 and mediate neuronal apoptosis in epilepsy [36]. Necrosis and apoptosis are two distinct types of mechanisms that mediate ischemic injury. Previous studies report that the expression of an exogenous p53 gene is sufficient for the induction of neuronal apoptosis [50–52]. However, the expression of exogenous p53 in the p53^−/−^ neurons alone in the absence of constitutively active DAPK1 (cDAPK1) does not cause apoptosis, showing that pS23 is an essential substrate of cDAPK1. Recently, Pei et al. found that activated DAPK1 phosphorylates p53 at serine-23 (pS23) via a direct binding of DAPK1 death domain (DAPK1DD) to the DNA-binding motif of p53 (p53DM), converging the signaling point of necrotic and apoptotic pathways in stroke [53]. They verified that the pS23 was located in both the mitochondria and the nucleus in the cultured cortical neurons. In the nucleus, pS23 induces the expression of proapoptotic genes, such as Bax and Puma, whereas in the mitochondrial matrix, pS23 triggers necrosis via interaction with cyclophilin D (CypD) in the cultured cortical neurons of mice (Fig. 3). Using yeast two-hybrid analysis and GST affinity binding assay, the authors confirmed that DAPK1DD is bound to a p53 DNA-binding motif consisting of amino acids 241–281 (p53DM^241–281^) [48]. The application of a synthesized membrane-permeable p53DM^241–281^ peptide (Tat-p53DM) that interrupts DAPK1-p53 interaction blocks these dual pathways of pS23 actions in vitro and in vivo [54]. Thus, the DAPK1-p53 interaction is a signaling point of convergence of necrotic and apoptotic pathways and is a desirable target for the treatment of ischemic insults.

### DAPK1 and Tau

**Fig. 4:**
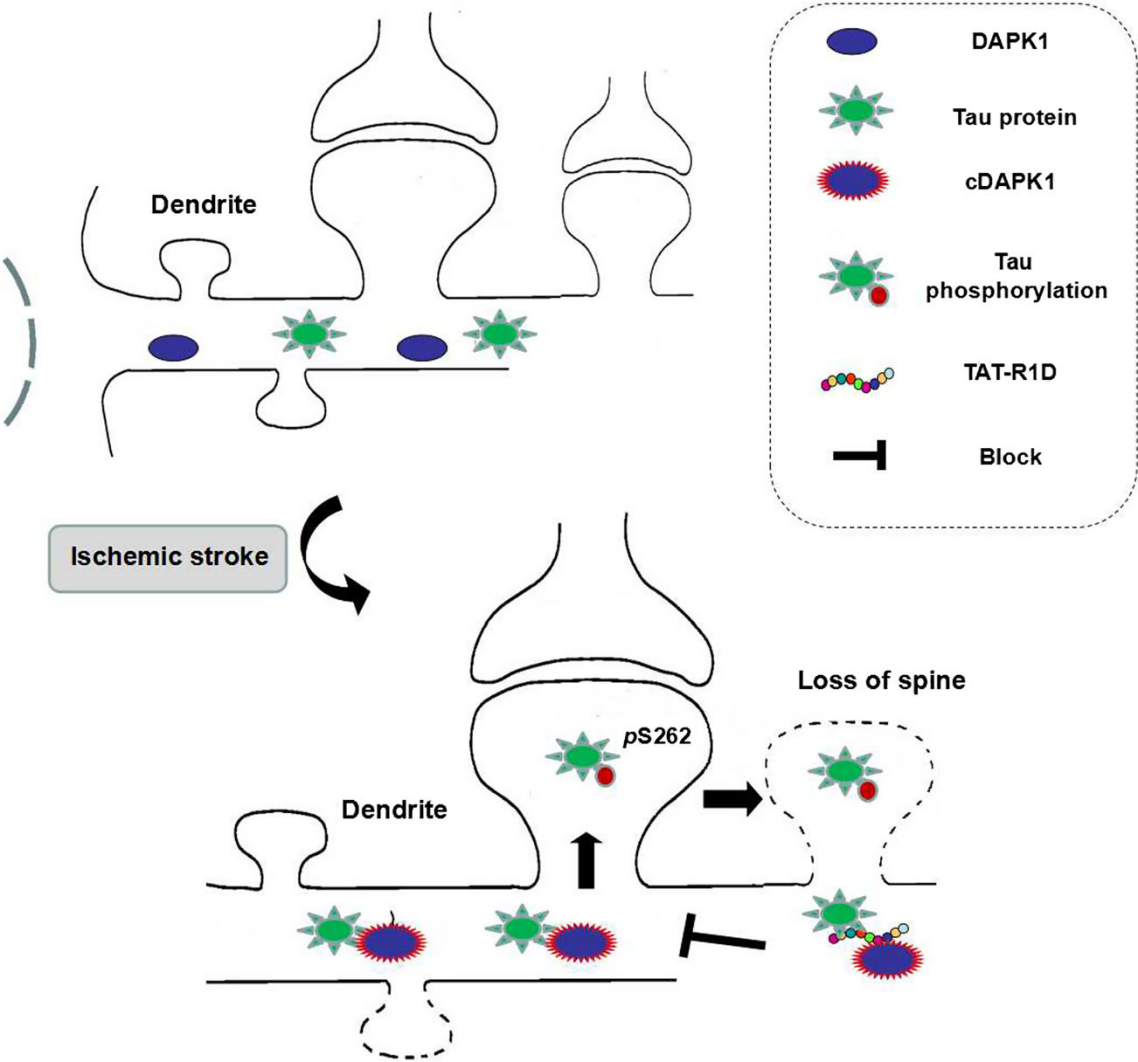
Illustration for DAPK1-Tau. DAPK1 is activated after cerebral ischemia and phosphorylates Tau Ser262, inducing spine loss and the following cell death. Blocking DAPK1-Tau interaction with a Tat-R1D peptide protects spine loss and ischemic stroke damage

Tau, abundantly expressed in the nervous system, plays a crucial role in neurodegenerative diseases such as Alzheimer’s disease. There are 79 potential serine and threonine phosphorylation sites on the longest human Tau isoform. Phosphorylation of these sites plays important roles in embryonic development, neurodegeneration, and ischemic stroke-induced neuronal death [55, 56]. The interaction between DAPK1 and Tau has been demonstrated in a few studies. DAPK1 could regulate microtubule synthesis, neuronal differentiation, and Tau toxicity by activating microtubules affinity kinase1/2 (MARK1/2) [57] and regulate the Tau protein stability by phosphorylating Pin1 (peptidylprolyl cis-trans isomerase 1) in serine 71 [58]. In cell experiments, DAPK1 interacts and phosphorylates Tau in threonine 231, serine 231, and serine 396. And in turn, the phosphorylated Tau can inhibit the death-promoting effect of DAPK1 [59]. Our recent study provides robust evidence supporting a causative role of Tau phosphorylation by DAPK1 in mediating dendritic spine injuries in ischemic stroke [60]. We showed that the activated DAPK1 phosphorylated Tau at Ser262 results in the possible formation of indissoluble tau and accumulation in the dendritic spines, which was the possible cause of synaptic damage in ischemic stroke. In addition, the deletion of DAPK1 kinase domain or blocking the interaction between DAPK1 and tau by a interfering peptide TAT-repeated 1 domain, IGSTENLK (TAT-R1D) distinctly reduced ischemia-induced spine loss and neuronal damage, indicating that intervening tau phosphorylation may be a target for the treatment of cerebral ischemia (Fig. 4).

### DAPK1 and Caytaxin

The caytaxin gene was identified in 2003, and its mutation could induce Cayman ataxia [61], which is a recessive congenital disorder associated with hypotonia, variable psychomotor retardation, cerebellar dysfunction like truncal ataxia and intention tremor, scoliosis, and ocular abnormalities [62]. Caytaxin is exclusively expressed in the adult and embryonic nervous system [63, 64]. Intriguingly, caytaxin was found to be present in the presynaptic cytosol, suggesting its possible role for presynaptic function [63].

Many studies reported that caytaxin plays an important role in axonal transporting [65], glutamate synthesis [66], synaptic apoptosis, and neurodegenerative diseases [67]. By using coimmunoprecipitation and mass spectrum detection, our unpublished preliminary results showed that DAPK1 could also interact and phosphorylate caytaxin on serine 46 at the presynaptic area in the mice model of middle cerebral artery occlusion (MCAO). How this presynaptic DAPK1-caytaxin interaction affects synaptic transmission and neuronal fate after ischemic stroke needs further exploration.

### DAPK1 and DANGER

DANGER, a partial MAB-21 domain-containing protein, identified on the basis of its binding to inositol 1, 4, 5-trisphosphate receptors (IP3R) [68], binds physiologically to DAPK1 [29]. Moreover, DANGER inhibits DAPK1 activity. Loss of DANGER’s inhibition of DAPK1 leads to enhanced cell death in the NMDA treatment and MCAO damage in vitro and in vivo respectively. Thus, drugs that enhance the inhibitory activity of DANGER on the DAPK1 signaling pathway might be useful in blocking cell death in stroke and neurodegenerative diseases.

## Conclusion

The pathophysiology and treatment of stroke remain daunting scientific and clinical problems. Despite impressive advances in elucidating the complexity of cell death mechanisms, the way forward may entail deciphering those intracellular signals that mediate cross-talk between DAPK1 and its downstream substrates involved in multiple pathways. Targeting DAPK1-related pro-death signals in both presynaptic and postsynaptic neurons may eventually lead us to improved methods to treat salvageable brain tissues after ischemic stroke.
